# Colchicine and Major Adverse Cardiovascular Events in Patients With Established Coronary or Cerebrovascular Atherosclerotic Disease: A Systematic Review and Meta-Analysis of Randomised Controlled Trials

**DOI:** 10.7759/cureus.114451

**Published:** 2026-08-12

**Authors:** Ahmed Alameleman Edris Ebrahim, Shaza Bakri Osman Elsayed, Nour Omera, Amal Hassan Sidahmed Ali, Areij Awad Osman Mohamed, Dalia Salih Mohammed Salih, Nibras Elfatih Hussein Abdalla, Esra Eltahir Mohamed Gamareldin

**Affiliations:** 1 Cardiology, NHS Foundation Trust, Angus, GBR; 2 Rheumatology, Croydon Health Services NHS Trust, London, GBR; 3 General Medicine, Royal Victoria Hospital, Belfast, GBR; 4 Internal Medicine, Elrazi University, Khartoum, SDN; 5 Cardiology, Cambridge University Hospitals NHS Foundation Trust, Cambridge, GBR; 6 Geriatrics, Walsall Healthcare NHS Trust, Walsall, GBR; 7 Pathology, School of Medicine, St. George’s University, St. George, GRD; 8 General Practice, Faculty of Medicine, Assiut University, Assiut, EGY

**Keywords:** atherosclerosis, colchicine, inflammation, major adverse cardiovascular events, meta-analysis, secondary prevention

## Abstract

Colchicine entered secondary-prevention guidelines on the strength of early randomised trials, but three large trials reported since 2024 have been neutral. We quantified its effect on major adverse cardiovascular events (MACE) and explored potential sources of heterogeneity between trials.

We searched PubMed/MEDLINE, Embase, Cochrane CENTRAL, Scopus, and trial registries to 30 June 2026 for randomised trials of oral colchicine versus placebo or usual care in adults with established coronary or cerebrovascular atherosclerotic disease, requiring at least three months of planned treatment and a composite MACE outcome. Risk ratios (RRs) were pooled using DerSimonian-Laird random-effects models; bias was appraised with RoB 2 and certainty with GRADE. All subgroup, meta-regression, and publication-bias analyses were regarded as exploratory.

Eight trials (30,392 participants) were included. MACE occurred in 1,138 of 15,199 patients receiving colchicine (7.5%) and 1,361 of 15,193 controls (9.0%): RR 0.75 (95% CI 0.62-0.89; p = 0.001), or 23 fewer events per 1,000 treated. Heterogeneity was substantial (I² = 76.7%), the 95% prediction interval (0.44-1.28) crossed unity, and the Hartung-Knapp-corrected interval reached the null (0.56-1.00). In exploratory analyses, estimates were smaller in larger trials (RR 0.85 vs 0.42) and in trials published in 2024-2025 (0.94 vs 0.59), and the funnel plot was asymmetric; these signals are correlated with one another and with population, design, and outcome definition, and cannot be attributed to any single characteristic. Restriction to trials at low risk of bias gave RR 0.84 (0.72-0.98). Certainty was low. Contemporary syntheses report no effect on all-cause mortality and an excess of gastrointestinal adverse events.

Colchicine reduced MACE overall, but the magnitude and generalisability of benefit remain uncertain, and benefit was less evident in larger and more recent trials. Confirmation in adequately powered contemporary trials, ideally with selection for residual inflammatory risk, is required.

## Introduction and background

Atherosclerotic cardiovascular disease remains the largest single contributor to premature mortality worldwide, and the risk that persists after an index coronary or cerebrovascular event is only partly explained by conventional risk factors. Even when low-density lipoprotein cholesterol, blood pressure, and platelet reactivity have been treated to contemporary targets, a substantial residual event burden remains. Much of that burden tracks with markers of systemic inflammation rather than with lipids, a phenomenon termed residual inflammatory risk: the excess of future cardiovascular events that is predicted by inflammatory biomarkers such as high-sensitivity C-reactive protein after lipid-lowering and antithrombotic therapy have been optimised [1]. This is biologically unsurprising because atherosclerosis is an immune-mediated disease in which endothelial activation, monocyte recruitment, and interleukin-1β-driven signalling govern plaque initiation, expansion, and destabilisation [2]. That inflammation is a modifiable determinant of outcome rather than merely a marker of arterial injury was established by the Canakinumab Anti-inflammatory Thrombosis Outcome Study, in which interleukin-1β neutralisation reduced recurrent cardiovascular events without any change in lipid concentrations [3]. Canakinumab is, however, parenteral, expensive, and associated with an excess of fatal infection, and the search for an inexpensive oral alternative has continued.

Colchicine is an attractive candidate. It binds tubulin dimers and disrupts microtubule polymerisation, thereby impairing neutrophil chemotaxis, adhesion, and degranulation and interfering with assembly of the NLRP3 inflammasome, the intracellular protein complex that converts pro-interleukin-1β to its active form and so initiates the interleukin-1 to interleukin-6 signalling axis implicated in plaque instability [4]; sterile crystalline and cholesterol stimuli within the plaque are recognised activators of this pathway [5]. Because these actions lie upstream of and are complementary to lipid lowering, colchicine offers a mechanistically distinct means of attenuating residual inflammatory risk at a fraction of the cost of biologic therapy, and at 0.5 mg daily, it is generally well tolerated apart from early gastrointestinal intolerance. Its narrow therapeutic index, renal and hepatic elimination, and susceptibility to interaction with cytochrome P450 3A4 and P-glycoprotein inhibitors nevertheless require that any population-level benefit be weighed against tolerability in the individual patient [6].

The clinical evidence has accumulated in two chronological phases whose results differ, although the trials also differ in population, timing of initiation, background therapy, follow-up, and outcome definition, so calendar time is only one of several correlated distinctions. The first low-dose colchicine trial in stable coronary disease reported a two-thirds reduction in acute coronary events [7]; the Colchicine Cardiovascular Outcomes Trial subsequently reported a 23% relative reduction after recent myocardial infarction [8], and the second low-dose colchicine trial a 31% reduction in chronic coronary disease [9]. On this basis, colchicine entered secondary-prevention guidelines, initially as a class IIb recommendation, meaning that treatment may be considered because usefulness is less well established, and subsequently as class IIa in the 2024 European Society of Cardiology chronic coronary syndromes guideline, meaning that treatment should be considered [10-12]; evidence syntheses of that era reported pooled risk reductions of 25%-35% [13]. Since 2024, however, three adequately powered trials have reported essentially neutral results: colchicine did not reduce recurrent vascular events after non-cardioembolic ischaemic stroke [14], did not reduce early stroke recurrence after minor stroke or high-risk transient ischaemic attack [15], and did not reduce the composite of cardiovascular death, recurrent myocardial infarction, stroke, or ischaemia-driven revascularisation when started soon after myocardial infarction [16]. These three trials together enrolled more than 18,000 patients. Contemporary meta-analyses have reached differing conclusions depending on their eligibility criteria and models [17-19].

We therefore undertook an updated systematic review and meta-analysis with two objectives: to estimate the effect of colchicine on major adverse cardiovascular events (MACE), defined as an investigator-adjudicated composite of cardiovascular death, myocardial infarction or acute coronary syndrome, stroke, and urgent coronary revascularisation, in patients with established coronary or cerebrovascular atherosclerotic disease; and to explore potential sources of heterogeneity between trials using prespecified subgroup analyses and meta-regression.

## Review

Materials and methods

Reporting, Registration and Ethics

This systematic review and meta-analysis was conducted and reported in accordance with the Preferred Reporting Items for Systematic Reviews and Meta-Analyses (PRISMA) 2020 statement [20]; the completed 27-item checklist is provided in the appendices. The review protocol was not prospectively registered in PROSPERO or any other registry. The eligibility criteria, primary outcome, subgroup analyses and meta-regression covariates described below were specified in an internal analysis plan agreed by the review team before data extraction began, but because that plan was not deposited in a public registry it cannot be independently verified; all subgroup, meta-regression and small-study analyses are therefore reported as exploratory rather than confirmatory throughout. Because the analysis used aggregate data from previously published trials and involved no contact with individual participants, institutional review board approval and informed consent were not required.

Eligibility Criteria

Eligibility followed the PICOS structure (Table 1). We included randomised controlled trials in adults with established atherosclerotic disease of the coronary or cerebral circulation, defined as chronic coronary syndrome, recent acute coronary syndrome or myocardial infarction, or recent non-cardioembolic ischaemic stroke or high-risk transient ischaemic attack. Eligible trials compared oral colchicine with placebo or usual care added to guideline-directed therapy, planned at least three months of treatment, and reported a composite MACE outcome with extractable event counts. The three-month threshold was set before data extraction because the hypothesis under test is sustained suppression of vascular inflammation rather than periprocedural anti-inflammatory action; it excluded single pre-procedural doses and short peri-infarct courses.

**Table 1:** Eligibility criteria (PICOS framework) PICOS: Population, Intervention, Comparator, Outcome, Study design; TIA: transient ischaemic attack; MACE: major adverse cardiovascular events; MI: myocardial infarction; ACS: acute coronary syndrome

Domain	Included	Excluded
Population	Adults ≥18 years with chronic coronary syndrome, recent acute coronary syndrome or myocardial infarction, or recent non-cardioembolic ischaemic stroke or high-risk TIA	Primary prevention; pericarditis; post-operative atrial fibrillation; cardio-embolic stroke
Intervention	Oral colchicine, any dose, added to guideline-directed therapy; planned duration ≥3 months	Single pre-procedural dose; courses <3 months; intravenous colchicine
Comparator	Matching placebo or usual care alone	Active comparator; non-randomised control
Outcome	Composite MACE (≥2 of cardiovascular death, MI or ACS, stroke, urgent revascularisation) with extractable event counts	Surrogate, biochemical or imaging endpoints only
Study design	Randomised controlled trial published in full; follow-up ≥3 months; no language restriction	Non-randomised designs; abstracts; preprints; reviews; secondary reports of included trials

Search Strategy, Study Selection and Data Extraction

We searched PubMed/MEDLINE, Embase, the Cochrane Central Register of Controlled Trials and Scopus from inception to 30 June 2026 without language restriction; all database searches were executed on 30 June 2026. The verbatim search string for each database, with the date run and the number of records returned, is provided in Appendix 1, and the registry search terms are given in the same table. ClinicalTrials.gov, the ISRCTN registry, the Australian New Zealand Clinical Trials Registry and the Chinese Clinical Trial Registry were searched for completed trials on the same date, and reference lists of included reports, previous reviews and guidelines were hand-searched with forward citation tracking in Google Scholar and Web of Science.

Records were screened by title and abstract, and then in full text, independently and in duplicate by two reviewers, with disagreements resolved by a third reviewer (κ = 0.89 at full-text stage). Data were abstracted onto a piloted form covering trial identity, design, blinding, population, allocation numbers, colchicine regimen and comparator, treatment and follow-up duration, the investigator-defined composite outcome and its components, and intention-to-treat event counts; all values were verified against the source publication by a second reviewer. Where a trial reported more than one composite, the one most closely matching the review definition was used; for CHANCE-3, whose primary outcome was recurrent stroke alone, the prespecified composite of vascular events was used [15]. A list of all reports excluded at full-text assessment, with the reason for exclusion in each case, is provided in Appendix 1. Risk of bias was assessed independently and in duplicate at the level of the primary outcome using version 2 of the Cochrane risk-of-bias tool (RoB 2), and overall judgements were derived using the RoB 2 algorithm, under which a judgement of high risk in any single domain yields an overall judgement of high risk [21].

Outcomes

The primary outcome was the investigator-defined composite of MACE, analysed by intention to treat. The constituent components differ meaningfully between trials, with some including all-cause rather than cardiovascular death, transient ischaemic attack, hospitalisation for unstable angina, or decompensated heart failure. Because this clinical heterogeneity is itself a candidate explanation for inconsistency, two prespecified outcome-definition sensitivity analyses were performed: a harmonised MACE analysis restricted to trials whose composite excluded all-cause death, transient ischaemic attack, and heart failure; and a core four-component analysis restricted to trials whose composite comprised only cardiovascular death, myocardial infarction, stroke, and coronary revascularisation. Mortality and safety outcomes are reported as a structured narrative synthesis, drawing on the individual trial reports and on contemporary quantitative syntheses, in the section "Mortality and safety outcomes."

Statistical Analysis

Analyses were performed in Python 3.12 using NumPy 2.4, SciPy 1.17, and pandas 3.0, and were independently reproduced in a second script written by a different author; the analysis code and the extracted dataset are provided as supplementary files. Risk ratios (RRs) were pooled on the log scale using inverse-variance random-effects models, with between-trial variance (τ²) estimated by the DerSimonian-Laird method of moments [22], and heterogeneity was quantified using Cochran's Q, τ², and I² [23]. Because the DerSimonian-Laird interval is anti-conservative when trials are few and heterogeneity is high, the estimate was recomputed with the Hartung-Knapp-Sidik-Jonkman correction, implemented with the ad hoc variance correction and t-distribution critical values on k - 1 degrees of freedom [24], and a 95% prediction interval was derived [25]; absolute effects were obtained by applying the pooled RR to the observed control-arm event rate. Six subgroup analyses (clinical setting, follow-up duration, trial design, trial size, publication period, and risk of bias) were compared using a chi-square test for subgroup differences, and univariable random-effects meta-regression, fitted by weighted least squares with τ² re-estimated from the residual Q statistic, examined publication year, log-transformed trial size, follow-up duration, and control-arm event rate. Robustness was assessed by leave-one-out and cumulative meta-analysis, fixed-effect and Peto models, the two outcome-definition analyses described above, and restriction to double-blind trials, trials at low risk of bias, trials excluding the one judged at high risk of bias, trials with ≥12 months of follow-up, trials with ≥500 participants, and coronary trials alone. Small-study effects were assessed by funnel plot and Egger's regression [26]. Two-sided p < 0.05 was considered significant, without adjustment for multiplicity, and certainty was rated using the GRADE framework [27]. Because only eight trials were available, the subgroup, meta-regression, and small-study analyses are underpowered and vulnerable to ecological confounding, and are reported and interpreted throughout as exploratory and hypothesis-generating.

Results

Study Selection

**Figure 1:**
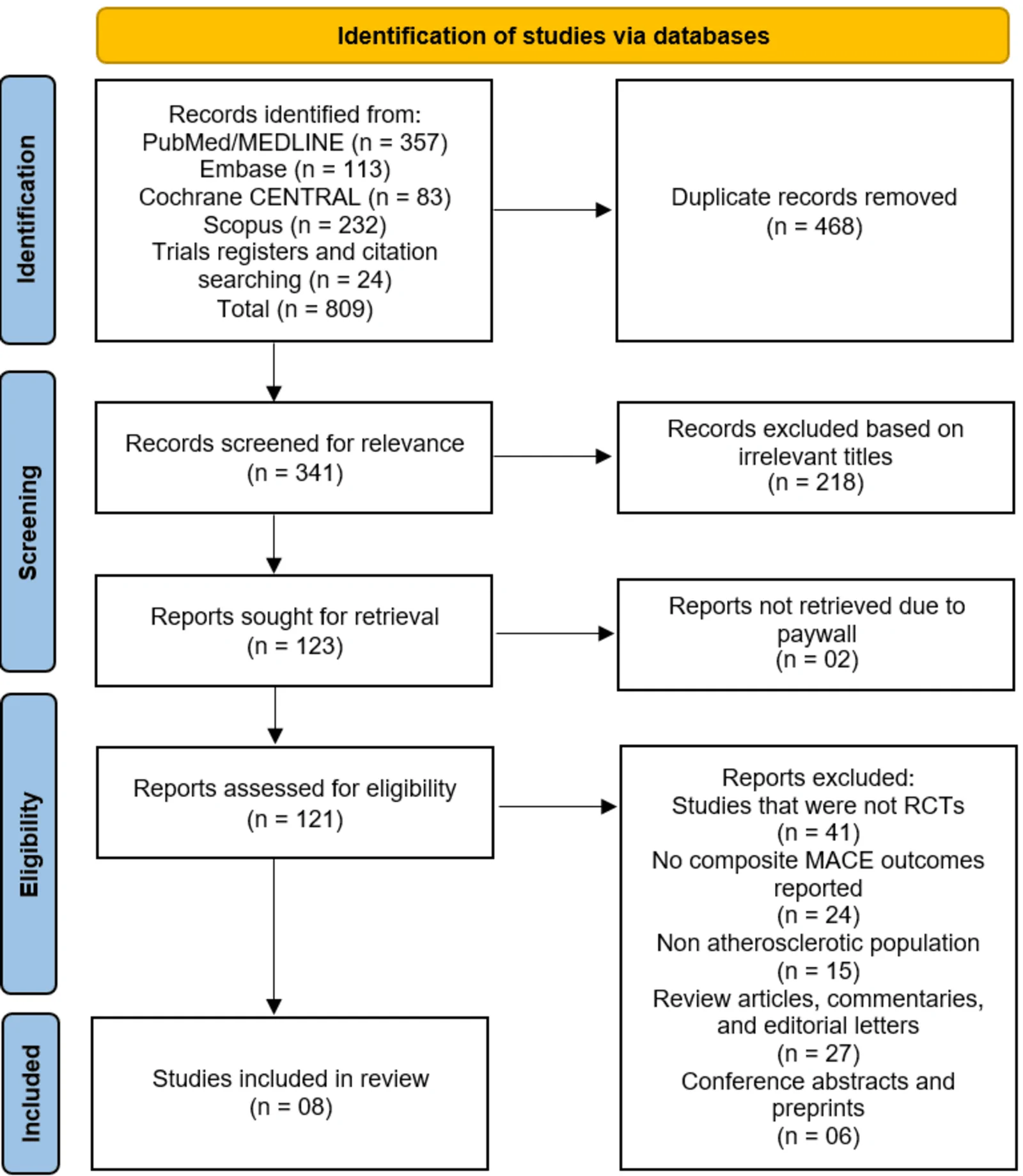
PRISMA 2020 flow diagram of study identification, screening and inclusion MACE: major adverse cardiovascular events

A total of 809 records were identified, comprising 357 from PubMed/MEDLINE, 113 from Embase, 83 from the Cochrane Central Register of Controlled Trials, 232 from Scopus, and 24 from trial registers and citation searching. After removal of 468 duplicates, 341 records were screened and 218 excluded on title and abstract. Of 123 reports sought for retrieval, two were unavailable because of paywall restrictions, leaving 121 full-text reports assessed for eligibility. Of these, 113 were excluded because they were not randomised controlled trials (n = 41), were reviews, commentaries, or editorial letters (n = 27), did not report a composite MACE outcome (n = 24), enrolled non-atherosclerotic populations (n = 15), or were conference abstracts or preprints (n = 6); these reports are itemised with reasons in Appendix 2. Eight randomised controlled trials met all eligibility criteria and were included: LoDoCo [7], COLCOT [8], COPS [28], LoDoCo2 [9], the trial reported by Akrami et al. [29], CONVINCE [14], CHANCE-3 [15], and CLEAR SYNERGY [16]. Study selection is summarised in Figure 1.

Characteristics of the Included Trials

The eight trials randomised 30,402 participants, of whom 30,392 (15,199 colchicine and 15,193 control) contributed to the intention-to-treat analysis; the 10 not analysed had withdrawn consent for use of their data in CONVINCE [14]. The trials were published between 2013 and 2025 across Australia, the Netherlands, Canada, Europe, South America, China and 14 further countries. Two enrolled patients with chronic coronary syndrome [7,9], four with recent acute coronary syndrome or myocardial infarction [8,16,28,29] and two with recent non-cardioembolic ischaemic stroke or high-risk transient ischaemic attack [14,15]. Six were double-blind and placebo-controlled; LoDoCo and CONVINCE used a prospective randomised open-label, blinded-endpoint design with a usual-care comparator. All but two used colchicine 0.5 mg once daily. Median follow-up ranged from 3 to 36 months, and background secondary-prevention therapy was intensive throughout, with antiplatelet use exceeding 93% and statin use exceeding 94% wherever reported. Characteristics are summarised in Table 2, and the composite outcomes and event data in Table 3.

**Table 2:** Characteristics of the eight included randomised controlled trials CONVINCE randomised 3,154 participants, of whom 3,144 were analysed. TIA: transient ischaemic attack; hs-CRP: high-sensitivity C-reactive protein; PCI: percutaneous coronary intervention

Trial (year)	Design and comparator	Population	N randomised (colchicine/control)	Colchicine regimen	Median follow-up
Nidorf et al. (2013) [7] (LoDoCo)	Open-label, blinded endpoint; no colchicine	Stable coronary disease on antiplatelet and statin therapy; Australia	532 (282/250)	0.5 mg once daily	36 months
Tardif et al. (2019) [8] (COLCOT)	Double-blind; placebo	Myocardial infarction within the preceding 30 days; 167 centres, 12 countries	4,745 (2,366/2,379)	0.5 mg once daily	22.6 months
Tong et al. (2020) [28] (COPS)	Double-blind; placebo	Acute coronary syndrome with angiographic coronary disease; 17 centres, Australia	795 (396/399)	0.5 mg twice daily for 1 month, then 0.5 mg daily	12 months
Nidorf et al. (2020) [9] (LoDoCo2)	Double-blind; placebo (after open-label run-in)	Chronic coronary disease, clinically stable ≥6 months; Australia and the Netherlands	5,522 (2,762/2,760)	0.5 mg once daily	28.6 months
Akrami et al. (2021) [29]	Double-blind; placebo	Acute coronary syndrome aged 40-70 years; single centre, Iran	249 (120/129)	0.5 mg once daily	6 months
Kelly et al. (2024) [14] (CONVINCE)	Open-label, blinded endpoint; usual care	Non-severe non-cardioembolic ischaemic stroke or high-risk TIA; 144 sites, Europe and Canada	3,154 (1,569/1,575)	0.5 mg once daily	34 months
Li et al. (2024) [15] (CHANCE-3)	Double-blind; placebo	Minor-to-moderate ischaemic stroke or high-risk TIA with hs-CRP ≥2 mg/L, within 24 h; 244 hospitals, China	8,343 (4,176/4,167)	0.5 mg twice daily for 3 days, then 0.5 mg daily	3 months
Jolly et al. (2025) [16] (CLEAR SYNERGY)	Double-blind, 2×2 factorial; placebo	Myocardial infarction within 72 h of index PCI (95% ST-elevation); 104 centres, 14 countries	7,062 (3,528/3,534)	0.5 mg once daily	36 months

**Table 3:** Investigator-defined composite outcome, event data and trial-level risk ratios

Trial	Composite outcome as defined by the investigators	Colchicine n/N (%)	Control n/N (%)	Risk ratio (95% CI)	Weight
Nidorf et al. (2013) [7] (LoDoCo)	Acute coronary syndrome, out-of-hospital cardiac arrest, or non-cardioembolic ischaemic stroke	15/282 (5.3)	40/250 (16.0)	0.33 (0.19-0.59)	6.6%
Tardif et al. (2019) [8] (COLCOT)	Cardiovascular death, resuscitated cardiac arrest, myocardial infarction, stroke, or urgent hospitalisation for angina leading to revascularisation	131/2,366 (5.5)	170/2,379 (7.1)	0.77 (0.62-0.97)	15.0%
Tong et al. (2020) [28] (COPS)	All-cause death, acute coronary syndrome, ischaemia-driven urgent revascularisation, or non-cardioembolic ischaemic stroke	24/396 (6.1)	38/399 (9.5)	0.64 (0.39-1.04)	7.9%
Nidorf et al. (2020) [9] (LoDoCo2)	Cardiovascular death, spontaneous myocardial infarction, ischaemic stroke, or ischaemia-driven coronary revascularisation	187/2,762 (6.8)	264/2,760 (9.6)	0.71 (0.59-0.85)	16.3%
Akrami et al. (2021) [29]	All-cause death, non-cardioembolic ischaemic stroke, hospitalisation for acute coronary syndrome, urgent revascularisation, or decompensated heart failure	8/120 (6.7)	28/129 (21.7)	0.31 (0.15-0.65)	4.5%
Kelly et al. (2024) [14] (CONVINCE)	Recurrent ischaemic stroke, myocardial infarction, cardiac arrest, or hospitalisation for unstable angina	153/1,569 (9.8)	185/1,575 (11.7)	0.83 (0.68-1.02)	15.6%
Li et al. (2024) [15] (CHANCE-3)	Ischaemic stroke, haemorrhagic stroke, transient ischaemic attack, myocardial infarction, or vascular death	298/4,176 (7.1)	309/4,167 (7.4)	0.96 (0.83-1.12)	17.0%
Jolly et al. (2025) [16] (CLEAR SYNERGY)	Cardiovascular death, recurrent myocardial infarction, stroke, or unplanned ischaemia-driven coronary revascularisation	322/3,528 (9.1)	327/3,534 (9.3)	0.99 (0.85-1.14)	17.2%
Pooled	-	1,138/15,199 (7.5)	1,361/15,193 (9.0)	0.75 (0.62-0.89)	100.0%

Risk of Bias

**Figure 2:**
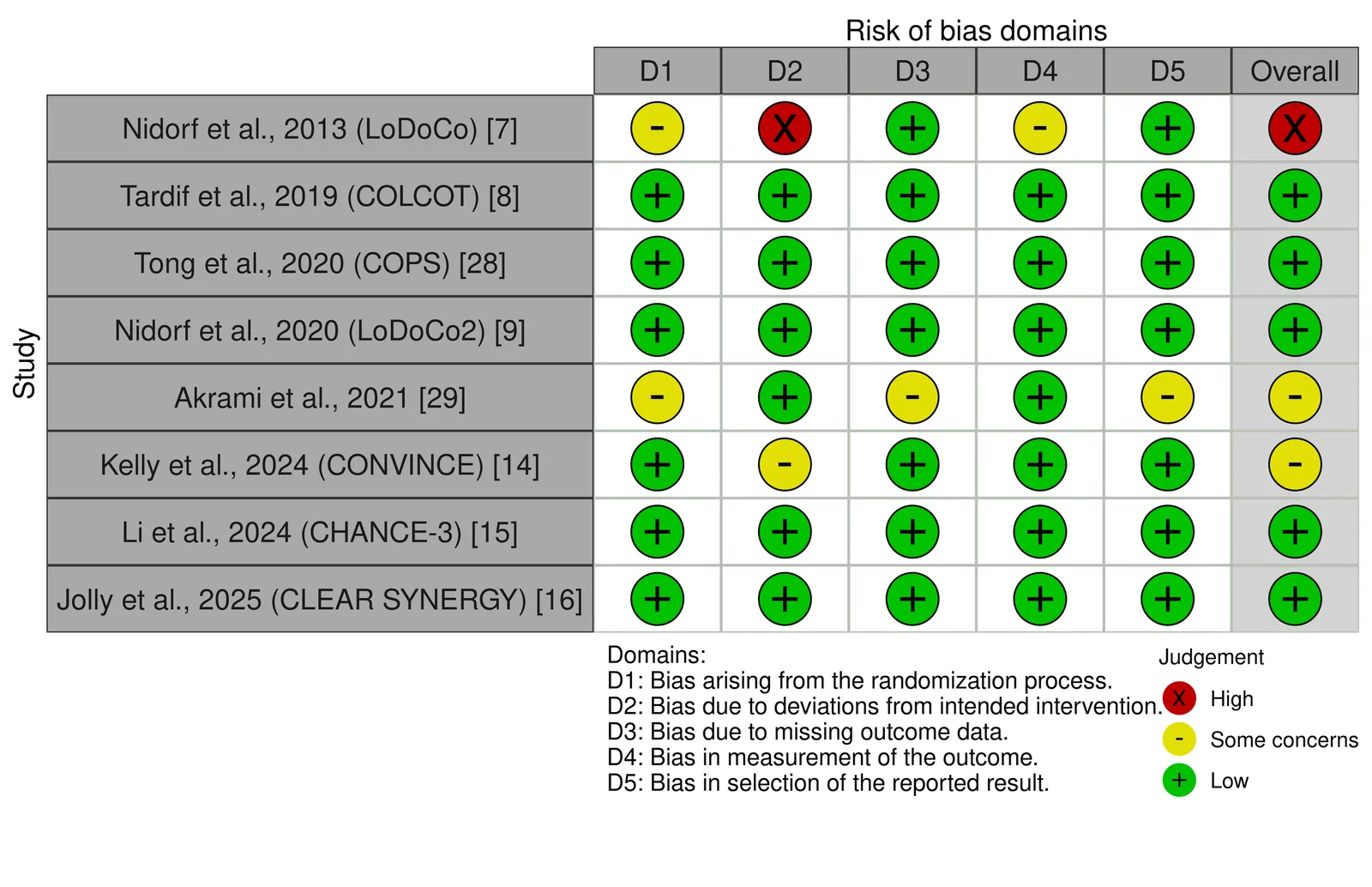
Risk-of-bias assessment using the Cochrane RoB 2 tool

Five trials were judged at low risk of bias overall, two raised some concerns and one was judged at high risk of bias overall (Figure 2). In LoDoCo, allocation was not centrally concealed, the trial was open-label with a no-treatment control, and 11% of patients allocated to colchicine withdrew within 30 days because of gastrointestinal intolerance; the domain covering deviations from the intended intervention was therefore rated at high risk, and under the RoB 2 algorithm a high-risk judgement in any domain yields an overall judgement of high risk [7,21]. We note that other syntheses have graded this trial as raising only some concerns [18]; we have applied the algorithm as published, and report a sensitivity analysis excluding this trial. In CONVINCE, the open-label design with a usual-care comparator raised concerns in the same domain, although endpoint adjudication was blinded [14]. In the trial by Akrami et al., the randomisation sequence and allocation concealment were incompletely described, no prospective analysis plan was available for comparison with the published report, and the number allocated to colchicine is reported inconsistently within the paper as both 120 and 122 [29]. The five remaining trials used central computerised randomisation with concealed allocation, maintained blinding of participants and outcome adjudicators, achieved near-complete outcome ascertainment, and reported outcomes consistent with their protocols.

Effect of Colchicine on MACE

**Figure 3:**
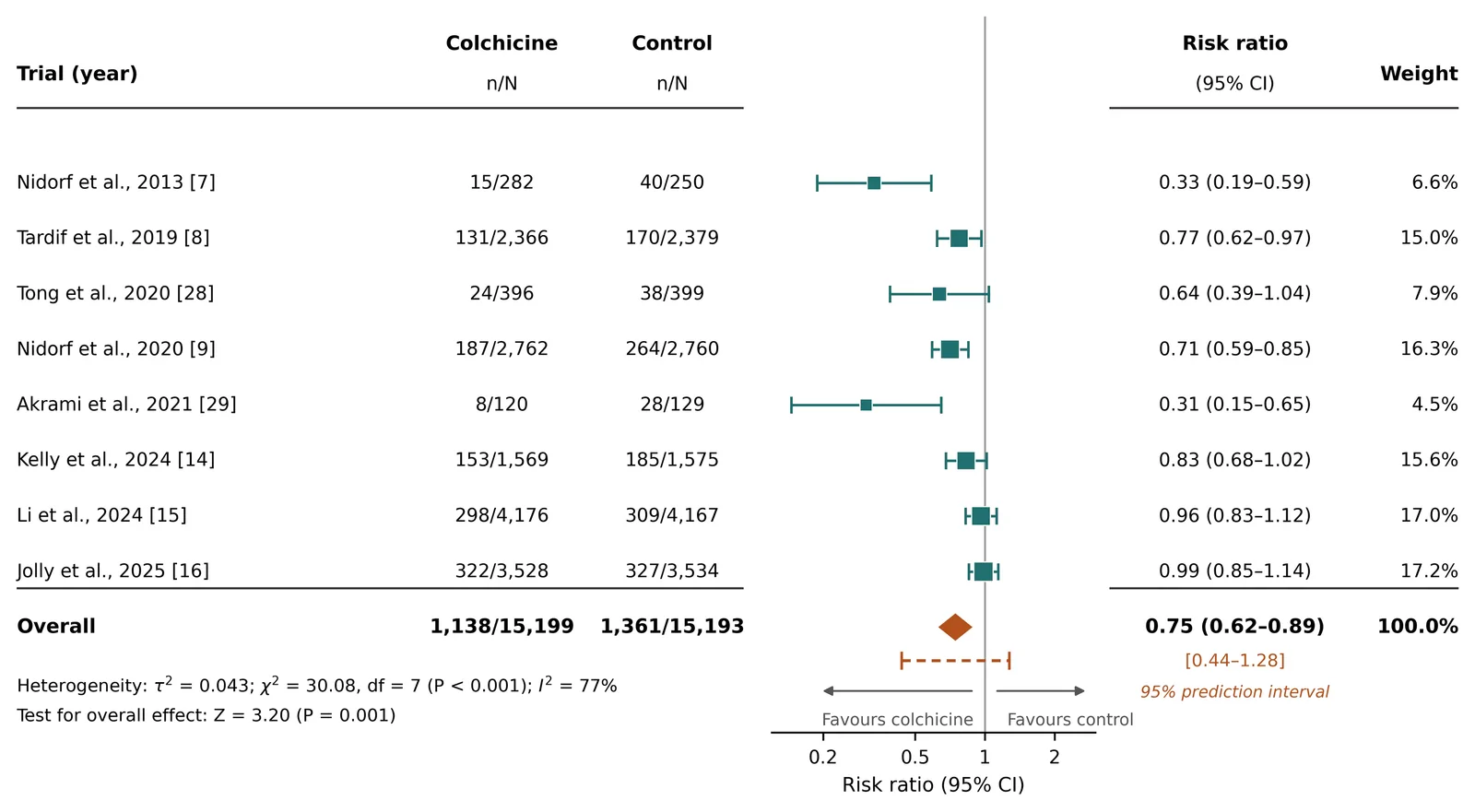
Forest plot of the effect of colchicine versus placebo or usual care on major adverse cardiovascular events, with reference numbers in square brackets Squares represent trial-level risk ratios with areas proportional to the random-effects weight; the diamond is the pooled DerSimonian-Laird estimate; the dashed bar beneath it is the 95% prediction interval.

A MACE occurred in 1,138 of 15,199 patients allocated to colchicine (7.5%) and 1,361 of 15,193 allocated to placebo or usual care (9.0%). In the random-effects model, colchicine was associated with a lower risk of MACE (RR 0.75, 95% CI 0.62-0.89; Z = 3.20; p = 0.001) (Figure 3 and Table 3). Applied to the pooled control-arm event rate of 9.0%, this corresponds to an absolute risk reduction of 2.3%, approximately 23 fewer events per 1,000 patients treated (95% CI 10 to 34 fewer), and a number needed to treat of 44 over a median follow-up of about two years.

Three findings qualify the precision and generalisability of this estimate. First, between-trial heterogeneity was substantial (τ² = 0.043; Cochran's Q = 30.08, df = 7, p < 0.001; I² = 76.7%), with trial-level RRs ranging from 0.31 to 0.99. Second, the 95% prediction interval was 0.44 to 1.28 and therefore included the null, indicating that the effect in a future comparable trial is not confidently predictable. Third, with the Hartung-Knapp-Sidik-Jonkman correction, the estimate was unchanged in magnitude, but its interval reached unity (RR 0.75, 95% CI 0.56-1.00; p = 0.050). The fixed-effect model, which weights the large contemporary trials more heavily, gave 0.84 (95% CI 0.78-0.91; p < 0.001), and the Peto odds ratio was 0.82 (95% CI 0.76-0.89). The primary estimate and the majority of sensitivity analyses therefore remained statistically significant, while the borderline Hartung-Knapp interval indicates that this significance is not robust to the choice of small-sample variance estimator.

Exploratory Subgroup Analyses

**Figure 4:**
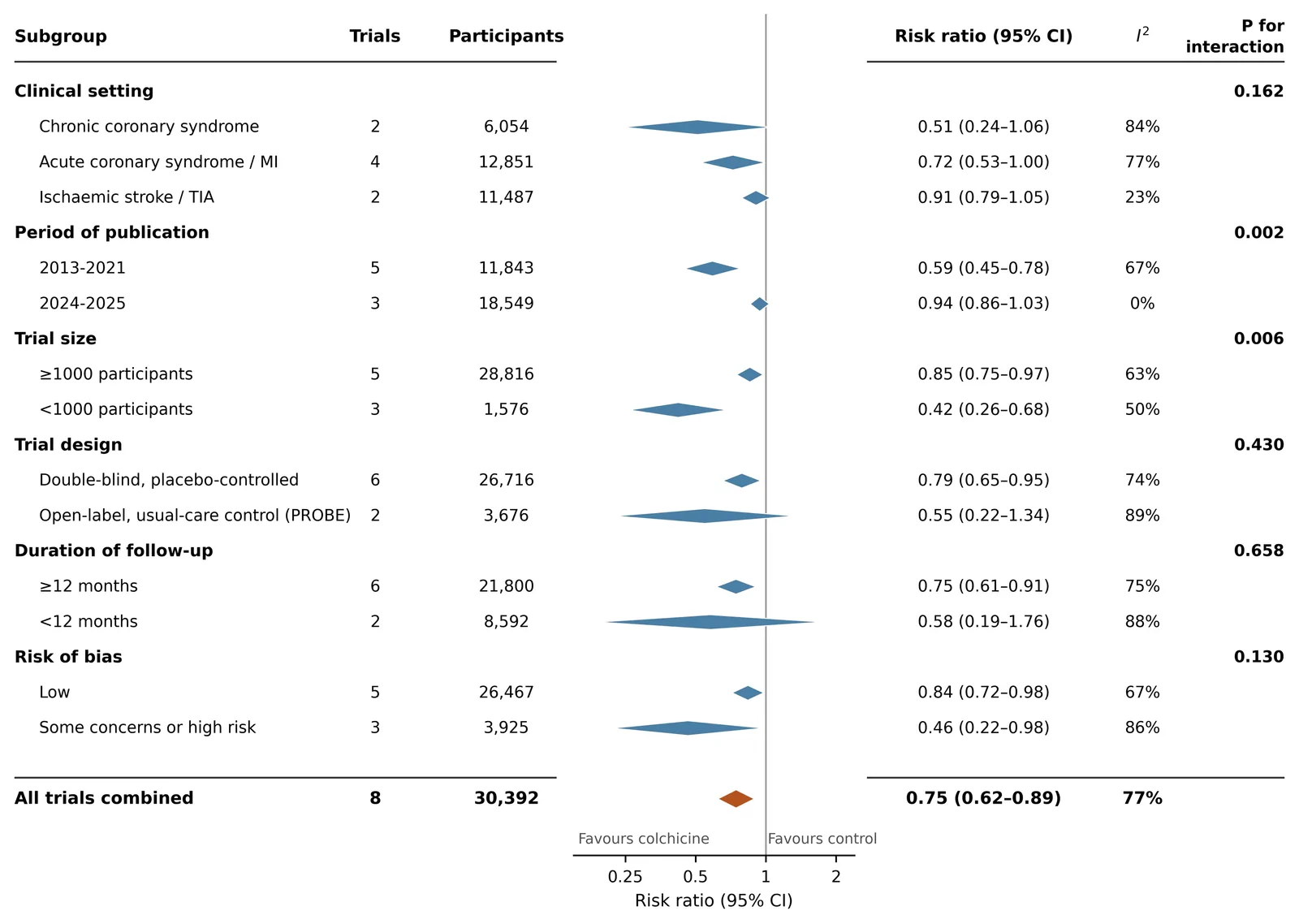
Exploratory subgroup analyses of the effect of colchicine on major adverse cardiovascular events Diamonds are random-effects pooled estimates within each subgroup; p-values for interaction are from the fixed-effect chi-square test for subgroup differences and are unadjusted for multiplicity.

The six subgroup analyses are shown in Figure 4 and Table 4. With only eight trials, these comparisons are underpowered, were not adjusted for multiplicity, and are presented as exploratory. The effect did not differ significantly by clinical setting: RR 0.51 (95% CI 0.24-1.06) in chronic coronary syndrome, 0.72 (95% CI 0.53-1.00) in acute coronary syndrome or myocardial infarction and 0.91 (95% CI 0.79-1.05) in ischaemic stroke or transient ischaemic attack (p for interaction = 0.162). Nor did it differ by duration of follow-up (≥12 months, RR 0.75; <12 months, RR 0.58; p = 0.658), trial design (double-blind, RR 0.79; open-label, RR 0.55; p = 0.430) or risk of bias (low, RR 0.84; some concerns or high, RR 0.46; p = 0.130).

**Table 4:** Exploratory subgroup analyses of the primary outcome TIA: transient ischaemic attack; MI: myocardial infarction

Subgroup	Trials	Participants	Risk ratio (95% CI)	I²	p for interaction
Clinical setting					0.162
Chronic coronary syndrome	2	6,054	0.51 (0.24-1.06)	84%	
Acute coronary syndrome or MI	4	13,051	0.72 (0.53-1.00)	77%	
Ischaemic stroke or TIA	2	11,487	0.91 (0.79-1.05)	23%	
Duration of follow-up					0.658
≥12 months	6	21,800	0.75 (0.61-0.91)	75%	
<12 months	2	8,592	0.58 (0.19-1.76)	88%	
Trial design					0.430
Double-blind, placebo-controlled	6	26,716	0.79 (0.65-0.95)	74%	
Open-label, usual-care control	2	3,676	0.55 (0.22-1.34)	89%	
Trial size					0.006
≥1,000 participants	5	28,816	0.85 (0.75-0.97)	63%	
<1,000 participants	3	1,576	0.42 (0.26-0.68)	50%	
Period of publication					0.002
2013-2021	5	11,796	0.59 (0.45-0.78)	67%	
2024-2025	3	18,596	0.94 (0.86-1.03)	0%	
Overall risk of bias					0.130
Low risk	5	25,140	0.84 (0.72-0.98)	67%	
Some concerns or high risk	3	5,252	0.46 (0.22-0.98)	86%	

Two comparisons were nominally discordant. Trials of fewer than 1,000 participants reported a pooled RR of 0.42 (95% CI 0.26-0.68) against 0.85 (95% CI 0.75-0.97) in larger trials (p for interaction = 0.006), and trials published between 2013 and 2021 reported 0.59 (95% CI 0.45-0.78) against 0.94 (95% CI 0.86-1.03) in the three trials published in 2024 and 2025, in which no heterogeneity was detected (I² = 0%; p for interaction = 0.002). These two subgroup variables are, however, strongly correlated with one another and with clinical setting, blinding, outcome definition and background therapy, so neither can be regarded as an independent modifier of effect.

Exploratory Meta-Regression

**Figure 5:**
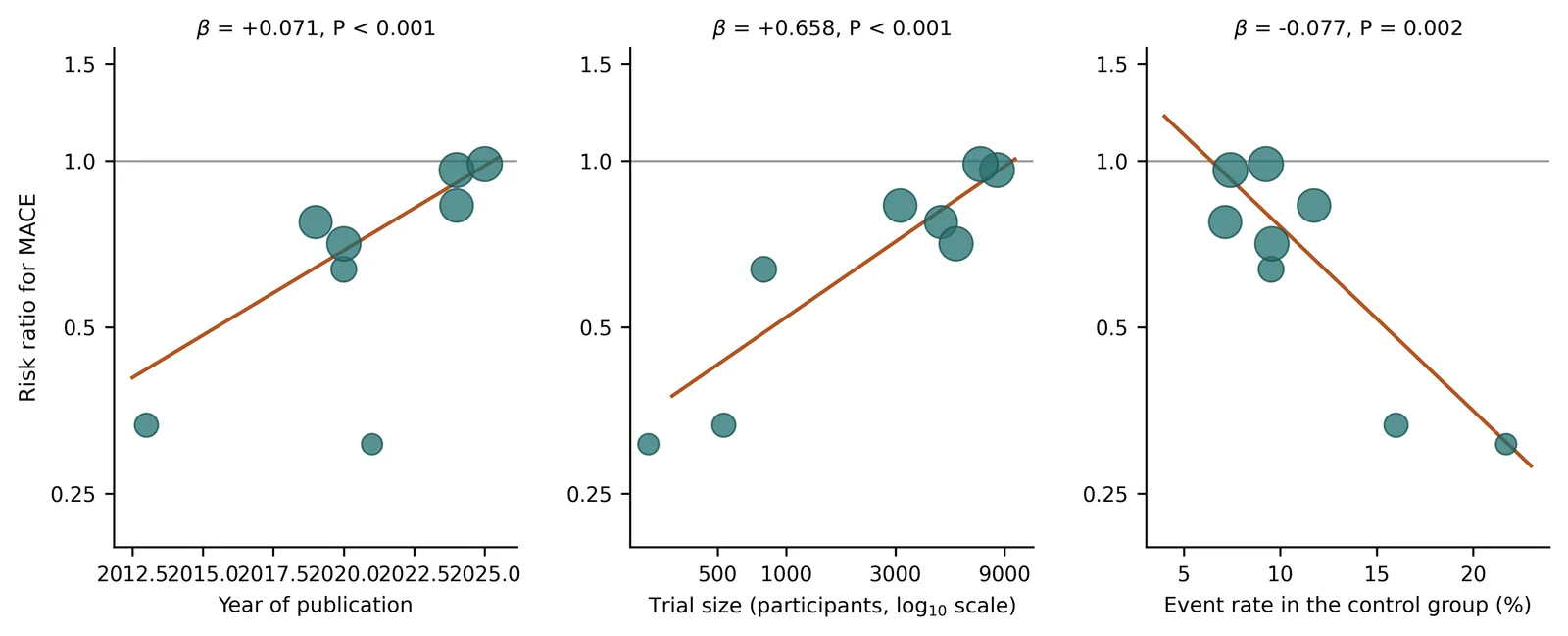
Exploratory random-effects meta-regression bubble plots for year of publication, trial size and control-arm event rate Bubble areas are proportional to the random-effects weight; the fitted line is the predicted risk ratio from the corresponding univariable model. Covariates are mutually correlated and models are not independent.

Meta-regression is shown in Figure 5. With eight trials and one covariate per model, these analyses are at high risk of overfitting and of ecological confounding, and the coefficients below should be read as descriptive summaries of trial-level association rather than as evidence that any characteristic causes the observed variation. Year of publication was associated with the log RR (β = +0.071 per year; SE 0.020; p < 0.001), and adding it to the model reduced residual between-trial variance from 0.043 to 0.009. Trial size showed a similar association (β = +0.658 per unit increase in log₁₀ sample size; SE 0.160; p < 0.001), reducing residual τ² to 0.006. With either covariate in the model, the residual Q statistic was no longer significant (p = 0.12 and p = 0.17). Because trial size and publication year are themselves highly correlated (later trials were larger), and both are correlated with population, blinding and outcome definition, these two models cannot be distinguished from one another or from a model based on any of the correlated clinical variables. The control-arm event rate was inversely associated with the log RR (β = -0.077 per 1% increase; SE 0.024; p = 0.002). Median duration of follow-up showed no association (β = +0.0005 per month; SE 0.0085; p = 0.954), and residual heterogeneity after adjustment for follow-up (τ² = 0.062) exceeded that of the unadjusted model.

Sensitivity Analyses and Robustness

**Figure 6:**
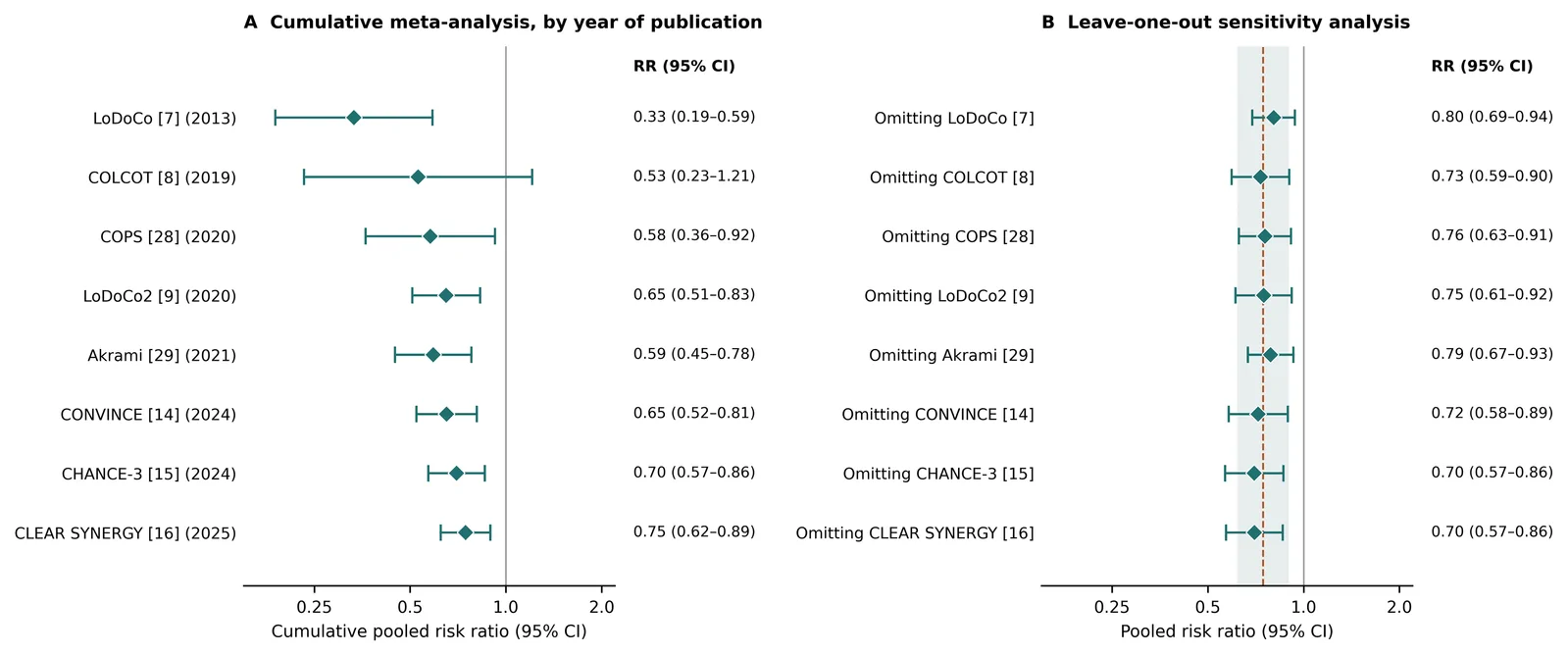
Cumulative and leave-one-out analyses (A) Cumulative meta-analysis ordered by year of publication. (B) Leave-one-out sensitivity analysis; the shaded band and dashed line indicate the confidence interval and point estimate of the primary analysis. Reference numbers are given in square brackets.

Sensitivity analyses are presented in Table 5, and the cumulative and leave-one-out analyses in Figure 6. The pooled estimate remained nominally significant in every analysis except the core four-component outcome analysis, but its magnitude varied. Restriction to double-blind trials attenuated it to 0.79 (95% CI 0.65-0.95), to trials at low risk of bias to 0.84 (95% CI 0.72-0.98), and to trials with at least 500 participants to 0.79 (95% CI 0.67-0.93). Excluding the single trial judged at high risk of bias gave 0.80 (95% CI 0.69-0.94; p = 0.006), confirming that the reclassification of LoDoCo does not overturn the primary finding. Restriction by follow-up duration left the estimate unchanged (≥12 months: 0.75, 95% CI 0.61-0.91). Excluding the two cerebrovascular trials strengthened it to 0.66 (95% CI 0.50-0.86).

**Table 5:** Sensitivity analyses of the primary outcome TIA: transient ischaemic attack; MACE: major adverse cardiovascular events; MI: myocardial infarction; CV: cardiovascular

Analysis	Trials	Risk ratio (95% CI)	p-value	I²
Primary analysis: DerSimonian-Laird random effects	8	0.75 (0.62-0.89)	0.001	76.7%
Hartung-Knapp-Sidik-Jonkman variance correction	8	0.75 (0.56-1.00)	0.050	76.7%
Fixed-effect (inverse variance) model	8	0.84 (0.78-0.91)	<0.001	76.7%
Peto odds ratio (fixed effect)	8	0.82 (0.76-0.89)	<0.001	-
Harmonised MACE (no all-cause death, TIA or heart failure)	5	0.76 (0.61-0.93)	0.009	79%
Core 4-component MACE (CV death, MI, stroke, revascularisation)	2	0.84 (0.61-1.16)	0.290	87%
Double-blind, placebo-controlled trials only	6	0.79 (0.65-0.95)	0.013	74%
Trials at low risk of bias only	5	0.84 (0.72-0.98)	0.027	67%
Excluding the trial at high risk of bias	7	0.80 (0.69-0.94)	0.006	69%
Trials with ≥12 months of follow-up only	6	0.75 (0.61-0.91)	0.003	75%
Trials with ≥500 participants only	7	0.79 (0.67-0.93)	0.004	72%
Excluding the two cerebrovascular trials	6	0.66 (0.50-0.86)	0.002	77%
Coronary trials with ≥12 months of follow-up only	5	0.71 (0.55-0.91)	0.008	78%

The two outcome-definition analyses addressed the clinical heterogeneity of the composite. Restricting to the five trials whose composite excluded all-cause death, transient ischaemic attack and heart failure gave a harmonised estimate of 0.76 (95% CI 0.61-0.93; p = 0.009), closely similar to the primary analysis, indicating that inclusion of these components does not by itself explain the pooled result. Restricting further to the two trials whose composite comprised only cardiovascular death, myocardial infarction, stroke and coronary revascularisation gave 0.84 (95% CI 0.61-1.16; p = 0.29); this analysis includes only LoDoCo2 and CLEAR SYNERGY and is too small to be informative, but it illustrates that the most strictly comparable outcome data are also the least conclusive.

In the leave-one-out analysis, the pooled RR ranged from 0.70 to 0.80 and remained significant throughout, so no single trial was responsible for the overall finding; heterogeneity remained substantial in every iteration (I² 69%-80%). In the cumulative meta-analysis, the pooled estimate was 0.33 (95% CI 0.19-0.59) after LoDoCo alone in 2013 and attenuated thereafter: 0.65 after LoDoCo2 in 2020, 0.59 in 2021, 0.65 after CONVINCE, 0.70 after CHANCE-3 and 0.75 after CLEAR SYNERGY. This pattern is consistent with regression of an early large estimate towards a smaller one as more, larger trials accrued, but it is equally consistent with the later trials having enrolled different populations under different conditions.

Exploratory Assessment of Small-Study Effects

**Figure 7:**
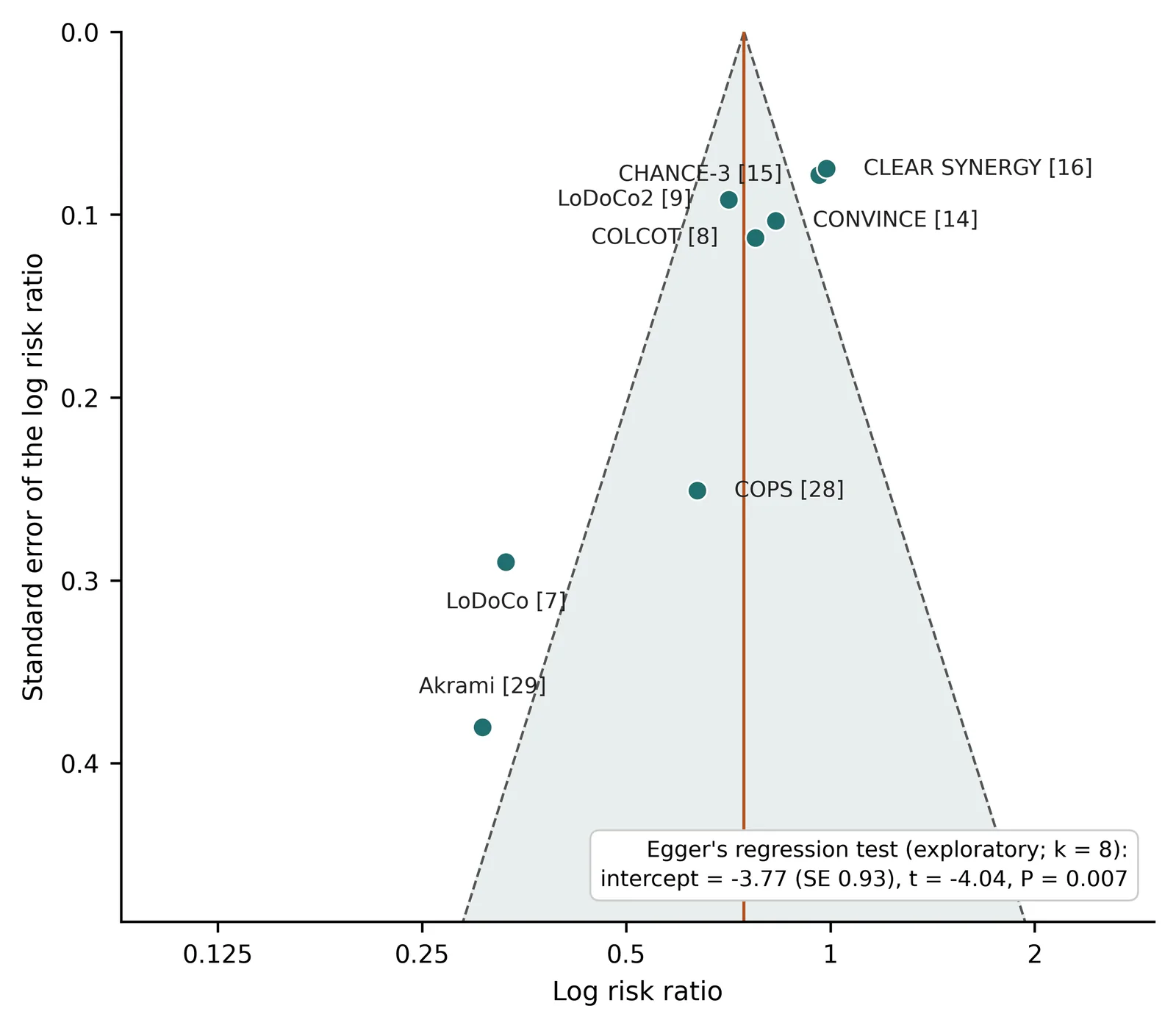
Funnel plot of the log risk ratio against its standard error, with reference numbers in square brackets The vertical line is the pooled random-effects estimate and the dashed diagonals delimit the pseudo 95% confidence region. Egger's test is exploratory given that fewer than 10 trials were available.

The funnel plot is shown in Figure 7. The three smallest trials lie towards the left of the plot and report the largest treatment effects, whereas the largest and most precise trials cluster around the null, and Egger's regression was statistically significant (intercept -3.77, SE 0.93; t = -4.04; p = 0.007). Egger's test is not recommended when fewer than 10 trials are available, because it has low power and an inflated false-positive rate under substantial heterogeneity, and this result must therefore be regarded as exploratory and interpreted with caution [26]. It is also notable that a contemporary meta-analysis of six long-term trials found symmetrical funnel plots and no significant Egger's test, illustrating how sensitive such assessments are to the set of trials included [18]. Asymmetry of the kind observed here may reflect selective publication of small positive trials, but is at least as likely to reflect methodological differences between small early trials and large contemporary ones, or genuine clinical differences between the populations studied.

Mortality and Safety Outcomes

Because our search and extraction were framed around the composite primary outcome, we did not undertake a de novo pooled analysis of the individual components; instead we summarise mortality and safety qualitatively from the included trial reports and quantitatively from two contemporary syntheses whose eligibility criteria overlap substantially with ours, so that the benefit-risk balance can be judged alongside the efficacy estimate. The 2025 Cochrane review of colchicine for secondary prevention, which pooled trials of at least six months' treatment, found moderate-certainty evidence of no effect on all-cause mortality (RR 1.01, 95% CI 0.84 to 1.21; 22,747 participants, 10 trials; I² = 1%) or on cardiovascular mortality (RR 0.94, 95% CI 0.73 to 1.22), no increase in serious adverse events (RR 0.98, 95% CI 0.94 to 1.02), and an increased risk of gastrointestinal adverse events, which were typically mild and transient [17]. A meta-analysis of six long-term trials reported concordant estimates for all-cause mortality (RR 1.01, 95% CI 0.80 to 1.28) and non-cardiovascular mortality (RR 1.08, 95% CI 0.76 to 1.54), with no excess of infection, pneumonia, hospitalisation for gastrointestinal events or cancer; in that analysis the reduction in the composite was driven by myocardial infarction, ischaemic stroke and urgent revascularisation, with no effect on cardiovascular death [18]. A further 2025 synthesis reported the same pattern of benefit on non-fatal ischaemic endpoints without a mortality signal [30].

At trial level, the safety findings that have attracted most attention are the numerical excess of non-cardiovascular death in LoDoCo2 and of total mortality in COPS, neither of which has been reproduced in the pooled analyses cited above [9,17,28]. Gastrointestinal intolerance is the dominant tolerability problem and the principal reason for discontinuation: 15.4% of patients screened for LoDoCo2 withdrew during the open-label run-in phase, principally because of gastrointestinal upset [9], 11% of patients allocated to colchicine in LoDoCo withdrew within 30 days for the same reason [7], and diarrhoea was significantly more frequent with colchicine in CLEAR SYNERGY and CHANCE-3 [15,16]. Taken together, the evidence indicates that colchicine does not reduce mortality, does not appear to increase serious adverse events, and does cause a clinically relevant excess of mild gastrointestinal symptoms sufficient to prevent a substantial minority of patients from continuing treatment.

Certainty of the Evidence 

The GRADE assessment is summarised in Table 6. Certainty began at high because all included studies were randomised trials. It was downgraded one level for inconsistency (I² = 76.7% with a prediction interval crossing the null) and a further level for imprecision, because the confidence interval reached the null once an appropriate small-sample variance correction was applied. It was not downgraded for risk of bias, because analyses restricted to trials at low risk of bias and excluding the trial at high risk of bias both remained significant, nor for indirectness. Publication bias was not used as a separate reason for downgrading, because with eight trials the formal test is unreliable and its result is not reproduced in other syntheses; it is instead noted as a possible contributor to inconsistency. Overall certainty is low, meaning that the true effect may differ substantially from the estimate.

**Table 6:** GRADE summary of findings

Outcome	Participants (trials)	Relative effect, RR (95% CI)	Anticipated absolute effects per 1,000 patients	Certainty	Reasons for downgrading
Major adverse cardiovascular events (investigator-defined composite)	30,392 (8 RCTs)	0.75 (0.62-0.89)	Control: 90 per 1,000; Colchicine: 67 per 1,000; Difference: 23 fewer (34 fewer to 10 fewer)	⊕⊕⊖⊖ Low	Serious inconsistency (I² = 76.7%; prediction interval 0.44-1.28); serious imprecision (Hartung-Knapp-corrected 95% CI 0.56-1.00 reaches the null). Possible small-study effects noted but not used as a separate reason for downgrading
All-cause mortality	22,747 (10 RCTs)	1.01 (0.84-1.21)	No important difference	⊕⊕⊕⊖ Moderate	Estimate taken from the 2025 Cochrane review rather than computed de novo in this review
Gastrointestinal adverse events	External synthesis	Increased with colchicine	Excess of mild, transient symptoms	⊕⊕⊕⊖ Moderate	Estimate taken from the 2025 Cochrane review rather than computed de novo in this review

Discussion

Principal Findings

In this systematic review and meta-analysis of eight randomised controlled trials enrolling 30,402 patients with established coronary or cerebrovascular atherosclerotic disease, colchicine was associated with a 25% relative reduction in MACE, equivalent to approximately 23 fewer events per 1,000 patients treated over a median of roughly two years. The primary estimate and most sensitivity analyses were statistically significant. The magnitude and generalisability of that benefit are, however, uncertain. Between-trial heterogeneity was substantial; the prediction interval for a future trial spanned both appreciable benefit and modest harm; and the confidence interval reached the null once the Hartung-Knapp-Sidik-Jonkman correction was applied, indicating that statistical significance depends on the choice of variance estimator. Benefit was less evident in larger and more recently published trials: the three trials reported since 2024, which together enrolled 18,596 patients and contributed roughly half of all events, produced a pooled RR of 0.94 (95% CI 0.86-1.03). The most defensible summary is therefore that colchicine probably reduces ischaemic events in some patients, that the size of that reduction is smaller than early trials suggested, and that the populations in whom it is worth pursuing have not been identified.

Interpretation of the Heterogeneity

Our exploratory analyses associated much of the between-trial variance with trial size and year of publication. We would caution strongly against reading this as a demonstration that either characteristic explains the discordance. With eight trials, meta-regression is at high risk of overfitting; trial size and publication year are almost perfectly correlated in this dataset, because the later trials were also the larger ones; and both are further correlated with clinical population, timing of initiation relative to the index event, blinding, comparator, background therapy and outcome definition. A trial-level association of this kind is vulnerable to ecological confounding and cannot separate these variables. What can reasonably be said is that the effect estimate is smaller in the trials that are individually most informative, and that no single clinical characteristic we examined accounts for the difference.

Several clinical explanations have been advanced since the neutral trials appeared, and the data neither confirm nor exclude them. The most frequently invoked is duration of exposure: the argument that anti-inflammatory therapy requires sustained administration before plaques stabilise, and that trials such as CHANCE-3 were too short [15]. This has precedent in the statin literature, where short-term trials in acute coronary syndromes were neutral while long-term trials were positive [31], and it underlies the threshold effect of cumulative colchicine exposure described by Li et al. [32]. In our data, follow-up duration showed no association with effect size (p = 0.954) and restriction to trials of at least 12 months left the estimate unchanged; CLEAR SYNERGY administered colchicine for a median of three years and was neutral [16]. Duration alone therefore does not reconcile the trials, though our analysis cannot exclude a threshold effect of achieved cumulative dose, which is not captured by planned follow-up. A second explanation is that colchicine is effective in coronary but not cerebrovascular disease [14,15]. The point estimate in cerebrovascular disease was closer to the null, and ischaemic stroke arises through diverse mechanisms of which large-artery atherosclerosis is only one; the CONVINCE investigators reported more favourable estimates in the on-treatment analysis and among patients with coexisting coronary disease [14]. The formal interaction test was not significant (p = 0.162), but with two cerebrovascular trials this test has little power, and excluding both trials leaves CLEAR SYNERGY, a purely coronary trial of 7,062 patients, neutral. A third explanation is that earlier estimates were inflated by the features that characterise small, early trials: less rigorous allocation concealment, open-label or subjectively assessed components, and sparse event numbers, all of which are empirically associated with larger apparent treatment effects [33,34]. LoDoCo reported a control-arm event rate of 16.0% over three years in stable coronary disease, roughly double that of LoDoCo2 in a comparable population, and the trial by Akrami et al. reported a six-month control event rate of 21.7% [7,29]. This explanation is consistent with the direction of our sensitivity analyses, in which restriction to low-risk-of-bias trials and use of the fixed-effect model both attenuated the estimate to 0.84, but it remains one hypothesis among several.

Comparison With Previous Syntheses

Syntheses conducted before the second wave of trials reported pooled reductions of 25%-35%, resting largely on LoDoCo, COLCOT and LoDoCo2 [13]. Contemporary syntheses that include the neutral trials have reached differing conclusions according to their eligibility criteria and models. Samuel et al., restricting inclusion to six trials with at least 12 months of follow-up and thereby excluding CHANCE-3, reported a 25% reduction in MACE with concordant reductions in myocardial infarction, ischaemic stroke and urgent revascularisation, and concluded that the evidence supports use of colchicine [18]; d'Entremont et al., using different endpoint definitions, reported broadly similar findings in the same issue [19]; and the 2025 Cochrane review, requiring at least six months of treatment, found benefit on myocardial infarction and stroke but moderate-certainty evidence of no effect on mortality or revascularisation [17]. Li et al., pooling 14 trials, reported a 28% reduction [32], and a further 2025 synthesis reported comparable estimates [30]. Our point estimate of 0.75 coincides with several of these. Where we differ is in emphasis rather than in arithmetic: as the accompanying editorial to the two European Heart Journal analyses observed, the apparent agreement between syntheses conceals genuine diversity in the underlying trials, and the pooled average is a summary of trials that differ in ways that matter clinically [35]. Our contribution is to quantify that diversity explicitly through prediction intervals, outcome-definition sensitivity analyses and exploratory investigation of trial-level modifiers, and to conclude that the average is a less reliable guide to any individual patient than its confidence interval alone would suggest.

Biological Plausibility

It would be a mistake to conclude that the inflammatory hypothesis of atherothrombosis has been refuted. That hypothesis rests on evidence far broader than the colchicine trials, most directly on CANTOS [3] and on mechanistic work showing that colchicine suppresses NLRP3 inflammasome assembly, neutrophil-endothelial interaction and downstream cytokine release [4,6]. Colchicine reduced C-reactive protein in CLEAR SYNERGY and CONVINCE, confirming target engagement even where outcomes were unchanged [14,16]. Experimental work suggesting particular benefit in clonal haematopoiesis of indeterminate potential raises the possibility that benefit is real but confined to a definable inflammatory phenotype [36]. A drug that engages its target and lowers a validated biomarker without reducing events in an unselected population is more parsimoniously interpreted as insufficiently targeted than as inactive; the residual inflammatory risk colchicine addresses may be a smaller fraction of total residual risk in contemporary, intensively treated populations than when the earlier trials were designed [1]. Commentators on CLEAR SYNERGY have noted that more than half its participants were enrolled during the COVID-19 pandemic, that a signal towards benefit was present in the pre-pandemic stratum, that achieved on-treatment C-reactive protein remained above 2 mg/L, and that it enrolled almost exclusively patients treated within hours of primary percutaneous coronary intervention [18,37]. These considerations are post-hoc but not implausible, and they reinforce the view that the neutral result of a single trial should not be over-interpreted any more than the positive result of a single small trial.

Implications for Practice

The following observations are our interpretation of how this evidence might inform practice; they extend beyond what the meta-analysis alone can establish, particularly because mortality, safety and the individual components of the composite were not synthesised de novo in this review, and they should be read as commentary rather than as a formal recommendation. Low-certainty evidence of benefit does not warrant abandoning colchicine, and the drug is inexpensive, orally administered and mechanistically distinct from existing secondary-prevention therapies. Equally, the current guideline recommendations, which range from class IIb in the 2021 European prevention guideline to class IIa in the 2024 chronic coronary syndromes guideline [10-12], rest on an evidence base whose most recent and largest members are neutral, and guideline committees may wish to revisit them. For the individual patient, the trade-off is a benefit of uncertain size against a real burden of gastrointestinal intolerance sufficient to prevent a substantial minority from continuing treatment, with no evidence of mortality benefit [17]. On present evidence, it seems reasonable to consider colchicine principally in patients with chronic coronary disease who experience recurrent events despite optimal guideline-directed therapy, who have preserved renal and hepatic function, who are not taking strong inhibitors of cytochrome P450 3A4 or P-glycoprotein, and with whom the uncertainty has been discussed explicitly [38]. The case for routine initiation in the acute phase after ST-elevation myocardial infarction, or in unselected patients after non-cardioembolic ischaemic stroke, is weaker, because the two largest and most directly applicable trials in those settings were neutral [15,16]. The most useful next step is not a further unselected trial but one enrolling patients with demonstrable residual inflammatory risk, reporting individual participant data so that effect modification can be examined at patient rather than trial level.

Limitations

This review has several limitations. It used study-level aggregate data, so effect modification by baseline inflammatory status, adherence or achieved cumulative exposure could not be examined, and all trial-level associations are vulnerable to ecological confounding. With only eight trials, the subgroup analyses, meta-regression and tests for funnel-plot asymmetry are underpowered, unadjusted for multiplicity and at risk of overfitting; they are hypothesis-generating only. The investigator-defined composite differed across trials in ways that are clinically meaningful, and although two outcome-definition sensitivity analyses were performed, the most strictly comparable analysis included only two trials. Mortality, the individual components of the composite and safety outcomes were not synthesised de novo but summarised from contemporary syntheses, so the benefit-risk statements in this review depend on the validity of those analyses. Two trials were open-label with a usual-care comparator, which may exaggerate effects for outcomes with any subjective component. Finally, the review was not prospectively registered, so the prespecified status of our analyses cannot be independently verified, and searches closed on 30 June 2026.

## Conclusions

Across eight randomised trials in 30,392 patients with established coronary or cerebrovascular atherosclerotic disease, colchicine reduced MACE by 25%, corresponding to approximately 23 fewer events per 1,000 patients treated. The magnitude and generalisability of this benefit remain uncertain: heterogeneity was substantial, the prediction interval crossed the null, and the confidence interval reached unity under a small-sample variance correction. Benefit was less evident in larger and more recently published trials, although the trials also differed in population, treatment timing and outcome definition, and the reasons for their divergence cannot be resolved from trial-level data. Contemporary syntheses indicate no effect on mortality and an excess of mild gastrointestinal adverse events. On low-certainty evidence, colchicine remains a biologically plausible and inexpensive adjunct to secondary prevention whose net benefit in unselected patients is not yet established. Adequately powered trials that enrol patients selected for demonstrable residual inflammatory risk, and that report individual participant data, are the priority.

## Appendices

Appendix 1

Reports excluded at full-text assessment, with reasons

One hundred and twenty-one full-text reports were assessed for eligibility, and 113 were excluded. The principal excluded reports are itemised below with the reason for exclusion in each case. The remaining excluded records fell into the summary categories shown in Figure 1 of the main manuscript and comprised further non-randomised studies, reviews, commentaries and editorial letters, secondary or post-hoc reports of already included trials, and conference abstracts. Full citations are given in place of reference numbers so that this appendix can be read independently of the main reference list.

**Table 7:** Reports excluded at full-text assessment, with reasons

Excluded report	Participants	Reason for exclusion
Raju NC, Yi Q, Nidorf M, Fagel ND, Hiralal R, Eikelboom JW. J Thromb Thrombolysis. 2012, 33:88-94. 10.1007/s11239-011-0637-y (COOL)	80	Treatment duration 30 days; primary outcome high-sensitivity C-reactive protein; no composite MACE outcome
Deftereos S, Giannopoulos G, Raisakis K, et al. J Am Coll Cardiol. 2013, 61:1679-1685. 10.1016/j.jacc.2013.01.055	196	Primary outcome: angiographic in-stent restenosis; surrogate endpoint
Akodad M, Lattuca B, Nagot N, et al. Arch Cardiovasc Dis. 2017, 110:395-402. 10.1016/j.acvd.2016.10.004 (COLIN)	44	Treatment duration 1 month; primary outcome peak C-reactive protein
Vaidya K, Arnott C, Martinez GJ, et al. JACC Cardiovasc Imaging. 2018, 11:305-316. 10.1016/j.jcmg.2017.08.013	80	Primary outcome: low-attenuation plaque volume on computed tomography; imaging endpoint only
Hennessy T, Soh L, Bowman M, et al. Am Heart J. 2019, 215:62-69. 10.1016/j.ahj.2019.06.003 (LoDoCo-MI)	237	Treatment duration 30 days; primary outcome high-sensitivity C-reactive protein
Bouabdallaoui N, Tardif JC, Waters DD, et al. Eur Heart J. 2020, 41:4092-4099. 10.1093/eurheartj/ehaa659	4,745	Time-to-treatment-initiation analysis of COLCOT; post-hoc report of an included trial
Shah B, Pillinger M, Zhong H, et al. Circ Cardiovasc Interv. 2020, 13:e008717. 10.1161/CIRCINTERVENTIONS.119.008717 (COLCHICINE-PCI)	400	Single pre-procedural dose; primary outcome percutaneous coronary intervention-related myocardial injury; follow-up 30 days
Mewton N, Roubille F, Bresson D, et al. Circulation. 2021, 144:859-869. 10.1161/CIRCULATIONAHA.121.056177 (COVERT-MI)	192	High-dose colchicine for 5 days only; primary outcome infarct size on cardiac magnetic resonance
Tong DC, Bloom JE, Quinn S, et al. Circulation. 2021, 144:1584-1586. 10.1161/CIRCULATIONAHA.121.054610	795	Two-year follow-up report of the COPS trial; secondary report of an included trial
Bouleti C, Viscogliosi S, Bresson D, et al. Open Heart. 2024, 11:e002474. 10.1136/openhrt-2023-002474	192	One-year follow-up report of COVERT-MI; treatment duration 5 days
Khiali S, Taban-Sadeghi M, Sarbakhsh P, Khezerlouy-Aghdam N, Entezari-Maleki T. Eur J Clin Pharmacol. 2024, 80:93-104. 10.1007/s00228-023-03582-5	106	Colchicine given only in combination with empagliflozin against differing empagliflozin regimens; primary outcomes: NYHA class and C-reactive protein

Appendix 2

Full electronic search strategy, dates and yield

All database searches were executed on 30 June 2026 by a single reviewer and independently re-run by a second reviewer on the same date. No language, date or publication-status restrictions were applied.

**Table 8:** Full electronic search strategy, dates and yield

Source	Search string	Date run	Records
PubMed/MEDLINE	("Colchicine"[Mesh] OR colchicine[tiab] OR colcrys[tiab] OR mitigare[tiab] OR gloperba[tiab]) AND ("Cardiovascular Diseases"[Mesh] OR "Coronary Disease"[Mesh] OR "Coronary Artery Disease"[tiab] OR "Coronary Heart Disease"[tiab] OR CAD[tiab] OR CHD[tiab] OR "Acute Coronary Syndrome"[Mesh] OR ACS[tiab] OR "Myocardial Infarction"[Mesh] OR myocardial infarction[tiab] OR MI[tiab] OR "Chronic Coronary Syndrome"[tiab] OR "Stable Coronary Disease"[tiab] OR "Ischemic Heart Disease"[tiab] OR "Atherosclerotic Cardiovascular Disease"[tiab] OR ASCVD[tiab] OR angina[tiab] OR "Percutaneous Coronary Intervention"[Mesh] OR PCI[tiab] OR stroke[Mesh] OR stroke[tiab]) AND ("Randomized Controlled Trial"[Publication Type] OR randomized[tiab] OR randomised[tiab] OR randomly[tiab] OR placebo[tiab] OR trial[tiab] OR RCT[tiab])	30 June 2026	357
Embase (Ovid)	('colchicine'/exp OR colchicine:ti,ab OR colcrys:ti,ab OR mitigare:ti,ab OR gloperba:ti,ab) AND ('cardiovascular disease'/exp OR 'coronary artery disease'/exp OR 'coronary heart disease':ti,ab OR CAD:ti,ab OR CHD:ti,ab OR 'acute coronary syndrome'/exp OR ACS:ti,ab OR 'myocardial infarction'/exp OR MI:ti,ab OR 'ischemic heart disease':ti,ab OR 'chronic coronary syndrome':ti,ab OR 'atherosclerotic cardiovascular disease':ti,ab OR ASCVD:ti,ab OR angina:ti,ab OR 'percutaneous coronary intervention'/exp OR PCI:ti,ab OR stroke/exp) AND ('randomized controlled trial'/exp OR random*:ti,ab OR placebo*:ti,ab OR trial:ti,ab OR RCT:ti,ab)	30 June 2026	113
Cochrane CENTRAL	(colchicine OR colcrys OR mitigare OR gloperba) AND ("coronary artery disease" OR "coronary heart disease" OR CAD OR CHD OR "acute coronary syndrome" OR ACS OR "myocardial infarction" OR MI OR "ischemic heart disease" OR "chronic coronary syndrome" OR "atherosclerotic cardiovascular disease" OR ASCVD OR angina OR PCI OR stroke OR "cardiovascular disease"). CENTRAL predominantly indexes randomized and quasi-randomized trials, so an RCT filter was not applied.	30 June 2026	83
Scopus	TITLE-ABS-KEY(colchicine OR colcrys OR mitigare OR gloperba) AND TITLE-ABS-KEY("cardiovascular disease" OR "coronary artery disease" OR "coronary heart disease" OR CAD OR CHD OR "acute coronary syndrome" OR ACS OR "myocardial infarction" OR MI OR "ischemic heart disease" OR "chronic coronary syndrome" OR "stable coronary disease" OR "atherosclerotic cardiovascular disease" OR ASCVD OR angina OR "percutaneous coronary intervention" OR PCI OR stroke) AND TITLE-ABS-KEY(random* OR placebo* OR trial OR "randomized controlled trial" OR RCT)	30 June 2026	232
ClinicalTrials.gov	Condition or disease: cardiovascular disease OR coronary artery disease OR myocardial infarction OR stroke; Intervention: colchicine; Study type: interventional (randomized); Status: completed or terminated with results	30 June 2026	14
ISRCTN registry	colchicine AND (cardiovascular OR coronary OR myocardial infarction OR stroke)	30 June 2026	3
ANZCTR	colchicine AND (cardiovascular OR coronary OR stroke)	30 June 2026	3
Chinese Clinical Trial Registry	colchicine (秋水仙碱) AND (cardiovascular OR coronary OR stroke)	30 June 2026	2
Citation searching	Forward and backward citation tracking of all included trials, previous systematic reviews and guideline documents in Google Scholar and Web of Science	30 June 2026	2
